# Supplementary material for: Distinct CSF biomarker-associated DNA methylation in Alzheimer’s disease and cognitively normal subjects
Source: Alzheimers Res Ther. 2023 Apr 10;15:78. doi: 10.1186/s13195-023-01216-7 (PMC10088180; doi:10.1186/s13195-023-01216-7)
Supplement: Supplementary file 2 — Additional file 2: Supplementary Figure 1. The estimated blood cell-type proportions from pre-mortem DNA methylation samples are not significantly associated with Braak scores measured on postmortem brain samples in the London dataset. Supplementary Figure 2. Overlap between significant individual CpGs (A) and significant DMRs (B) in the analysis of samples from AD subjects and cognitively normal subjects. Abbreviations: AD = Alzheimer's disease, CN = cognitivelynormal. Supplementary Figure 3. Overlap between significant individual CpGs (P < 10-5) and CpGs in significant DMRs (Sidak adjusted P < 0.05) associated with CSF biomarkers in (A) Alzheimer's disease subjects and (B) cognitively normal subjects. Supplementary Figure 4. The correlations of estimated effect sizes for CpG-to-CSF biomarker associations in cognitively normal (CN) subjects vs. those in Alzheimer's disease (AD) subjects are modest and non-significant. Supplementary Figure 5. The P-values for pathway enrichment of CSF biomarker-associated DNA methylation in cognitively normal (CN) samples are independent of those in Alzheimer's disease (AD) samples. Shown are 95 pathways (Supplementary Table 9) that reached FDR < 0.25 in either CN sample analysis or AD sample analysis. Abbreviations: R = Spearman correlation, p = P-value. Supplementary Figure 6. Immune cell type proportions estimated using the EpiDISH (PMID: 28193155) and the IDOL (labeled as "estimateCell") (PMID: 29843789) algorithms are highly concordant. Supplementary Figure 7. A subset of CpGs located in DMR chr7: 27183946 – 27184668 at HOXA5 geneshowed both (A) Significant association with Braak stage in London dataset (information extracted fromSupplementary Table 18). (B) Significant correlation between brain DNAm and blood DNAm. Supplementary Figure 8. DNA methylation (DNAm) at the DMR chr7:27183946 - 27184668 is significantly correlated with HOXA5 gene expression. The ADNI dataset with matched gene expression and DNAm measured on 263 subje [file 13195_2023_1216_MOESM2_ESM.pdf]

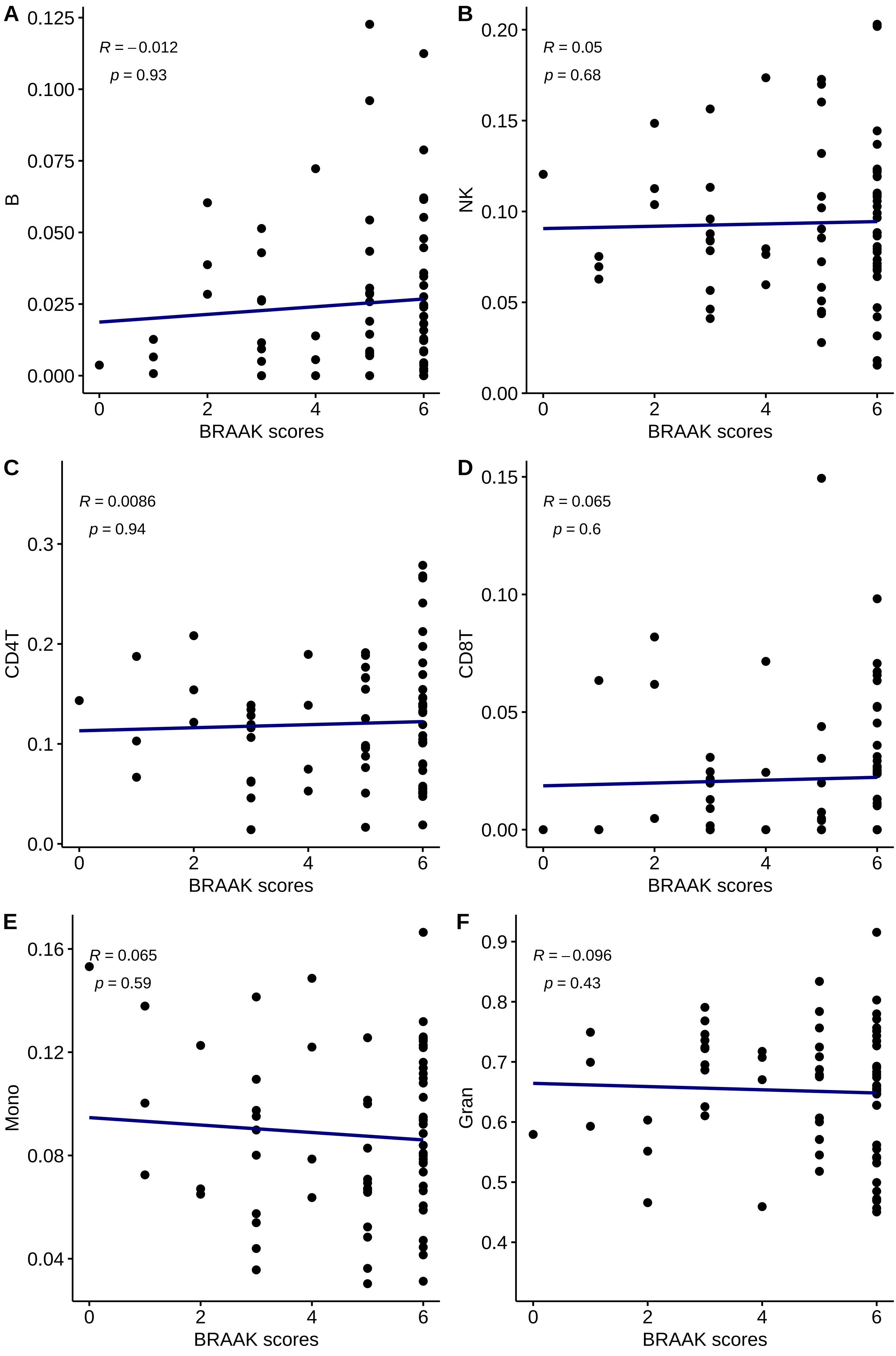

Supplementary Figure 1 The estimated blood cell-type proportions from pre-mortem DNA methylation samples are not significantly associated with Braak scores measured on postmortem brain samples in the London dataset.

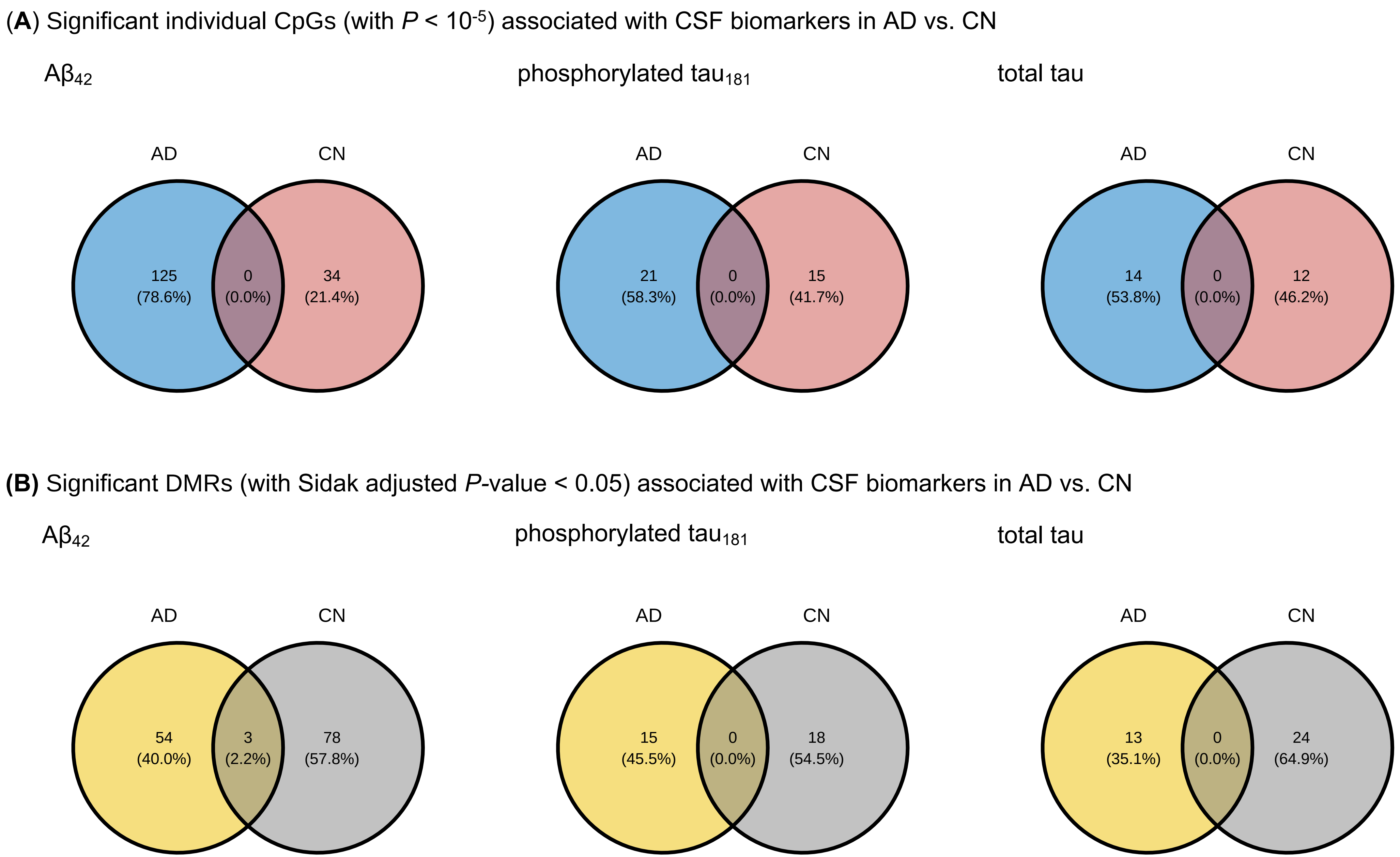

Supplementary Figure 2 Overlap between significant individual CpGs **(A)** and significant DMRs **(B)** in the analysis of samples from AD subjects and cognitively normal subjects. **Abbreviations:** AD = Alzheimer's disease, CN = cognitively normal

(A) Overlap of significant CpGs vs. CpGs in DMRs associated with CSF biomarkers in AD subjects

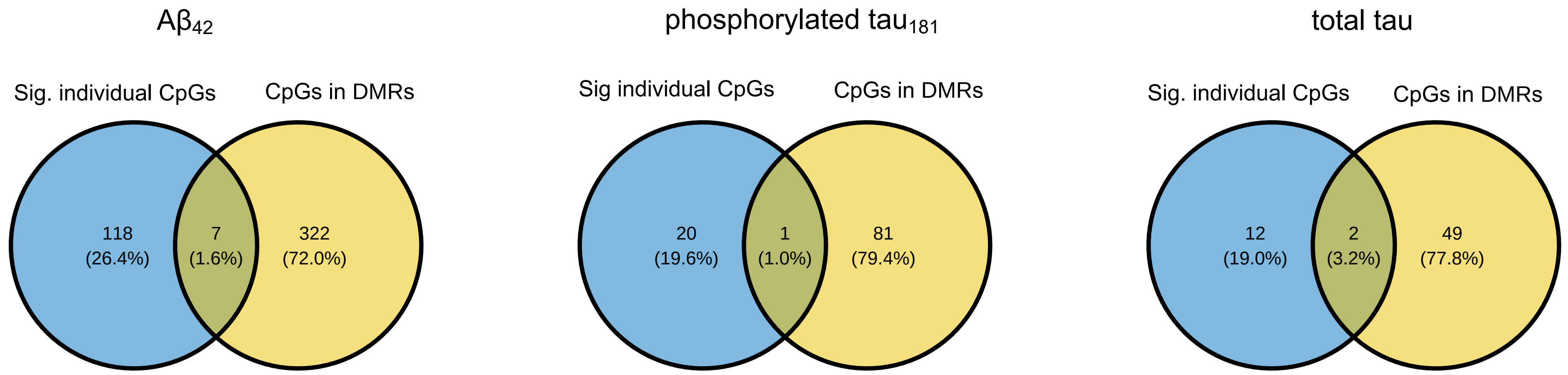

(B) Overlap of significant CpGs vs. CpGs in DMRs associated with CSF biomarkers in cognitively normal subjects

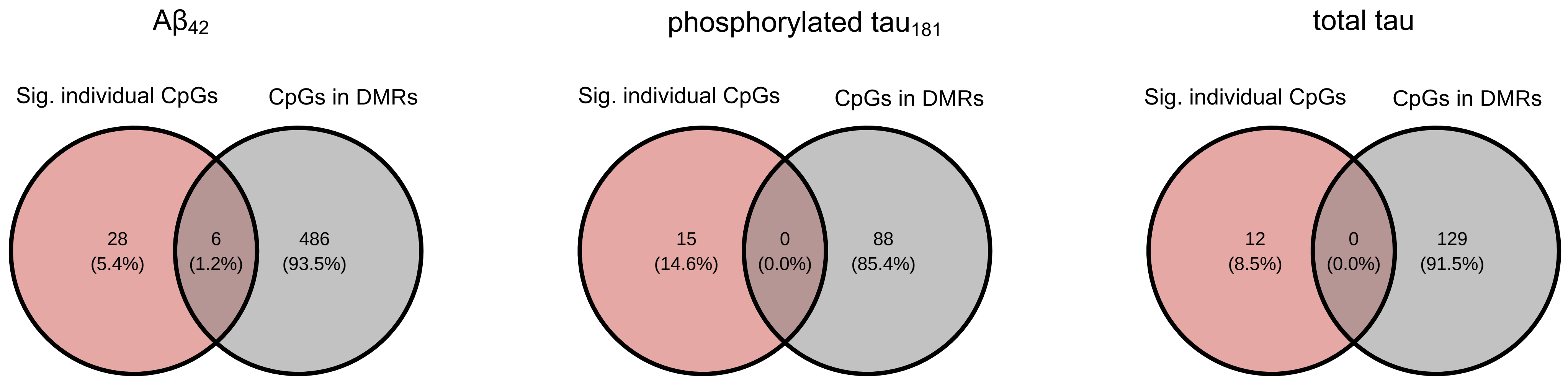

Supplementary Figure 3 Overlap between significant individual CpGs ($P < 10^{-5}$) and CpGs in significant DMRs (Sidak adjusted $P < 0.05$) associated with CSF biomarkers in (**A**) Alzheimer's disease subjects and (**B**) cognitively normal subjects.

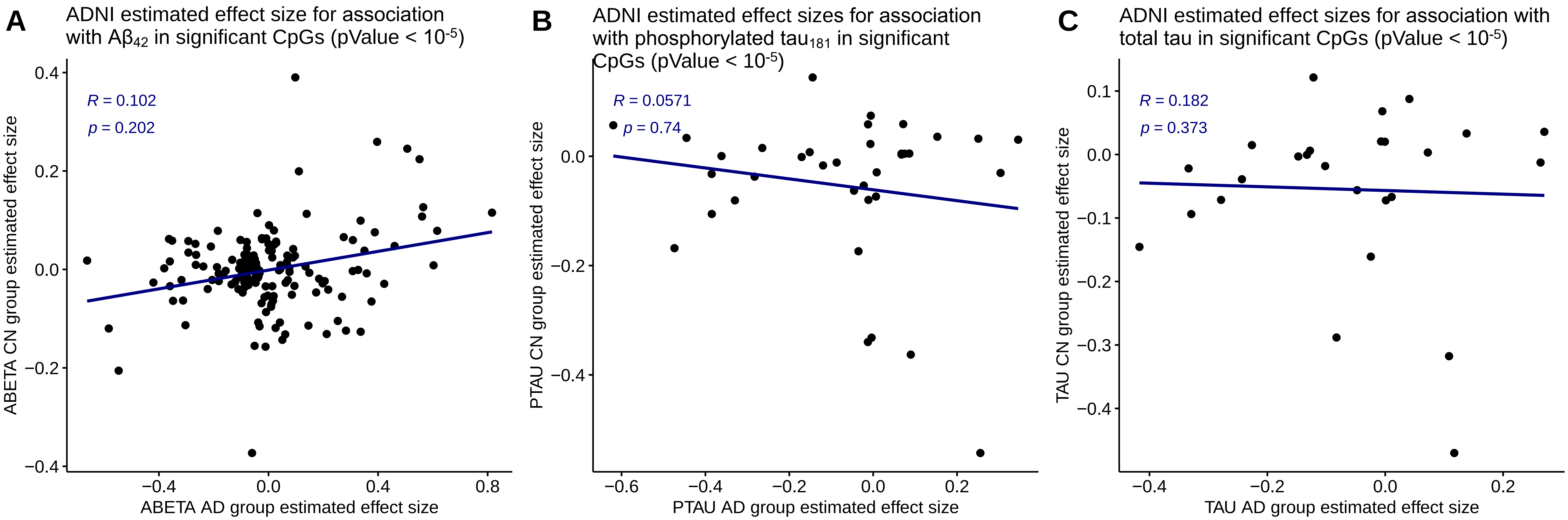

Supplementary Figure 4 The correlations of estimated effect sizes for CpG-to-CSF biomarker associations in cognitively normal (CN) subjects vs. those in Alzheimer's disease (AD) subjects are modest and non-significant.

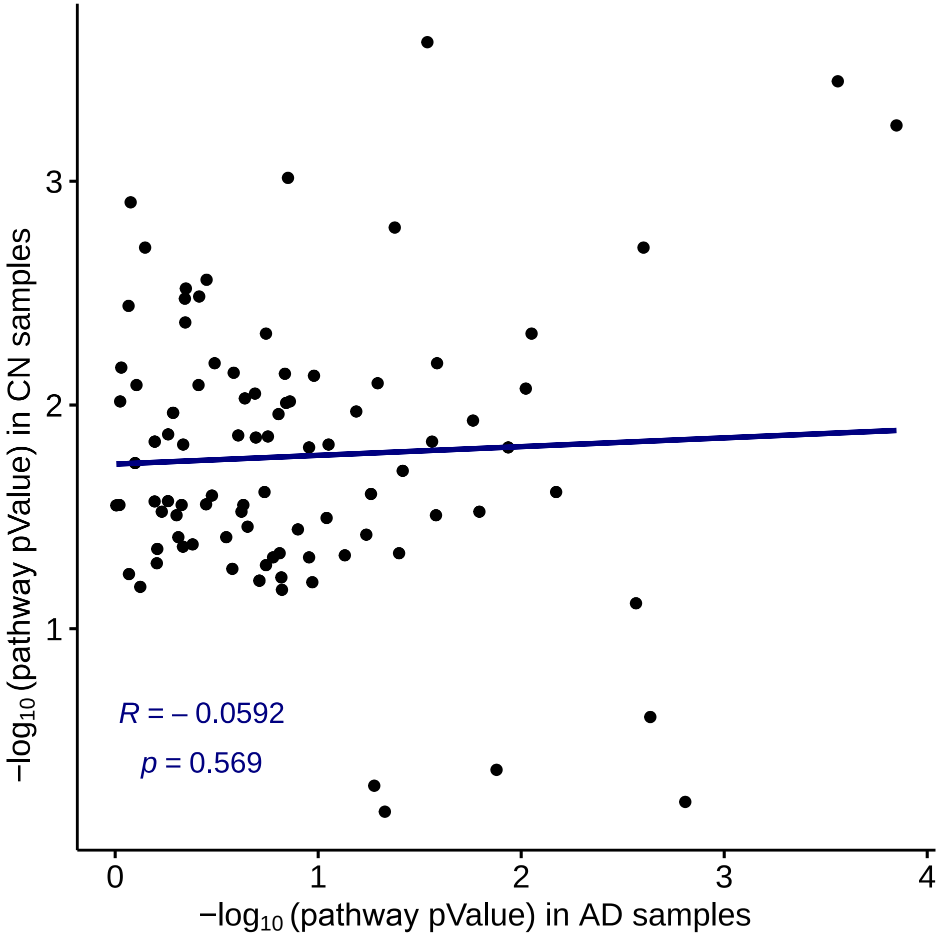

Supplementary Figure 5 The *P*-values for pathway enrichment of CSF biomarker-associated DNA methylation in cognitively normal (CN) samples are independent of those in Alzheimer's disease (AD) samples. Shown are 95 pathways (Supplementary Table 9) that reached FDR < 0.25 in either CN sample analysis or AD sample analysis. **Abbreviations**: *R* = Spearman correlation, *p* = *P*-value

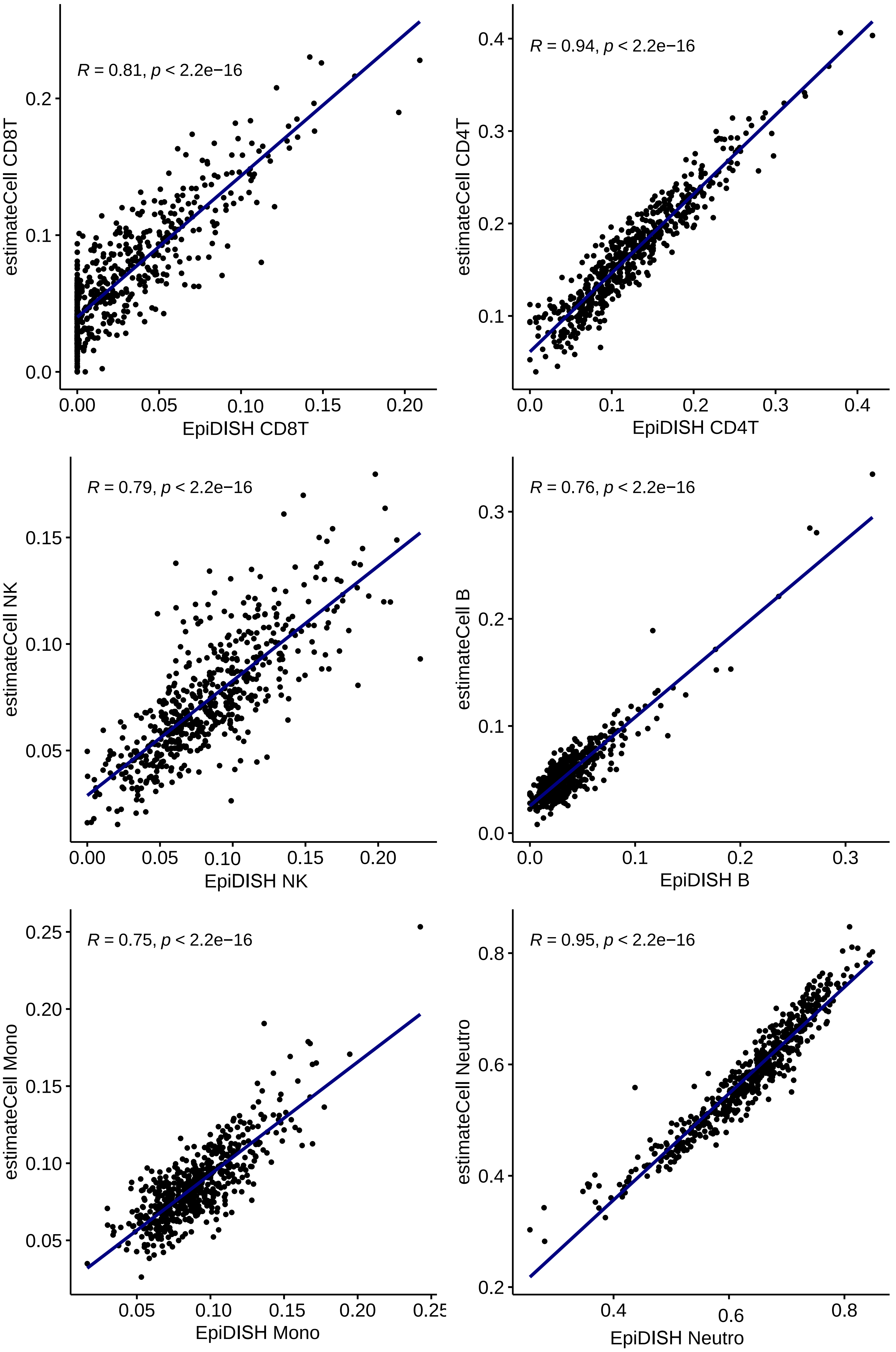

Supplementary Figure 6 Immune cell type proportions estimated using the EpiDISH (PMID: 28193155) and the IDOL (labeled as "estimateCell") (PMID: 29843789) algorithms are highly concordant.

Supplementary Figure 7 A subset of CpGs located in DMR chr7: 27183946 – 27184668 at *HOXA5* gene showed both **(A)** Significant association with Braak stage in London dataset (information extracted from Supplementary Table 18). **(B)** Significant correlation between brain DNAm and blood DNAm.

A Association of blood DNAm with Braak stage in London dataset

Significant CpGs in the analysis of London dataset (Replication)					
CpG	chr	position	estimate	se	pValue
cg14013695	chr7	27184176	0.384	0.169	2.34E-02
cg05774699	chr7	27184316	0.367	0.161	2.24E-02
cg26023912	chr7	27184369	0.698	0.275	1.13E-02
cg14882265	chr7	27184375	0.792	0.340	2.00E-02
cg07049592	chr7	27184450	0.422	0.201	3.61E-02
cg02106682	chr7	27184461	0.376	0.170	2.69E-02
cg03368099	chr7	27184521	0.492	0.202	1.49E-02

B. Brain DNAm vs. blood DNAm correlation in London dataset. Shown are methylation beta value meaured on postmortem brain prefrontal cortex and premortem whole blood from samples in the London dataset.

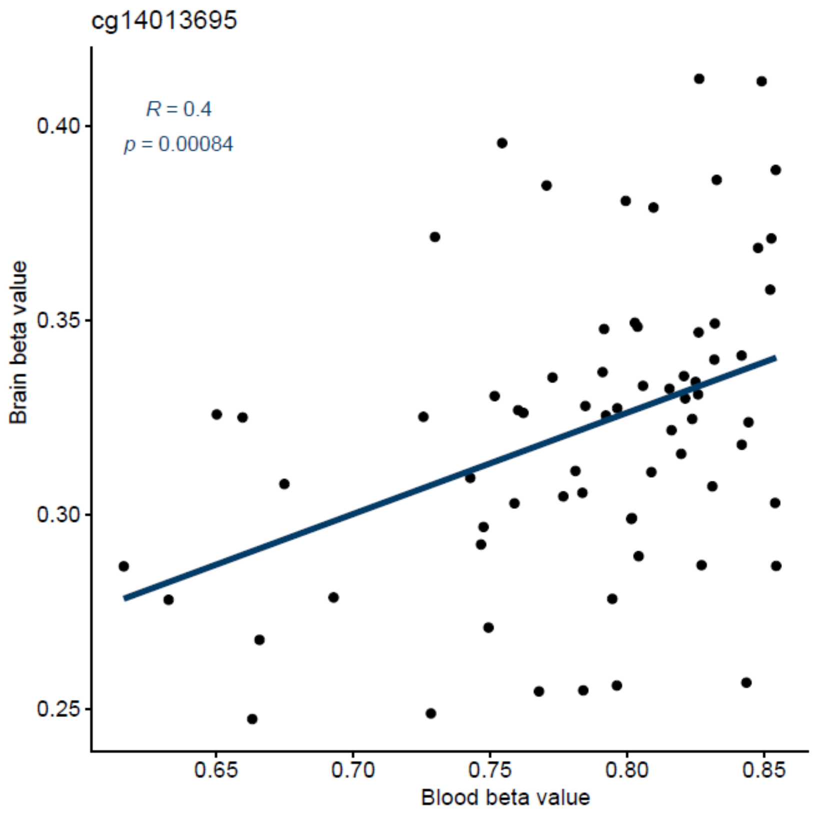

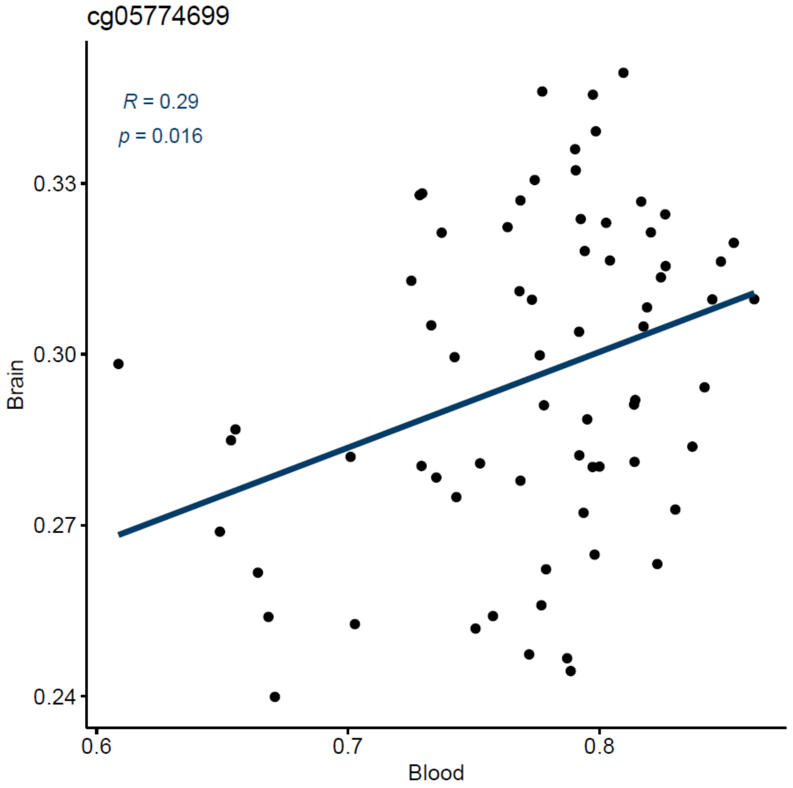
cg05774699
R = 0.29
p = 0.016
0.33
0.30
0.27
0.24
Brain
0.6
0.7
0.8
Blood

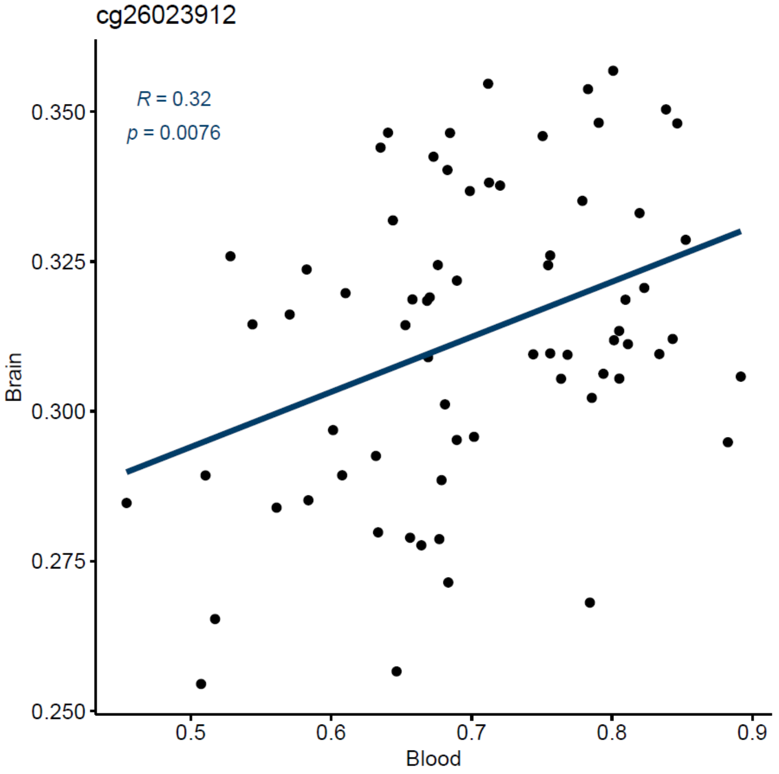
cg26023912
R = 0.32
p = 0.0076
0.350
0.325
0.300
0.275
0.250
Brain
0.5
0.6
0.7
0.8
0.9
Blood

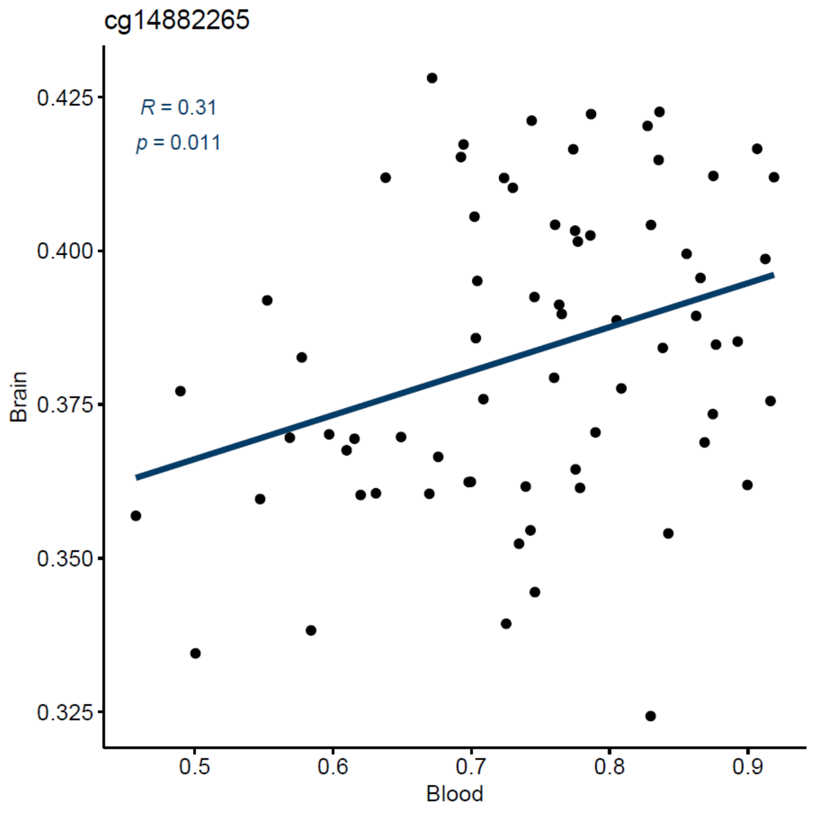
cg14882265
R = 0.31
p = 0.011
0.425
0.400
0.375
0.350
0.325
Brain
0.5
0.6
0.7
0.8
0.9
Blood

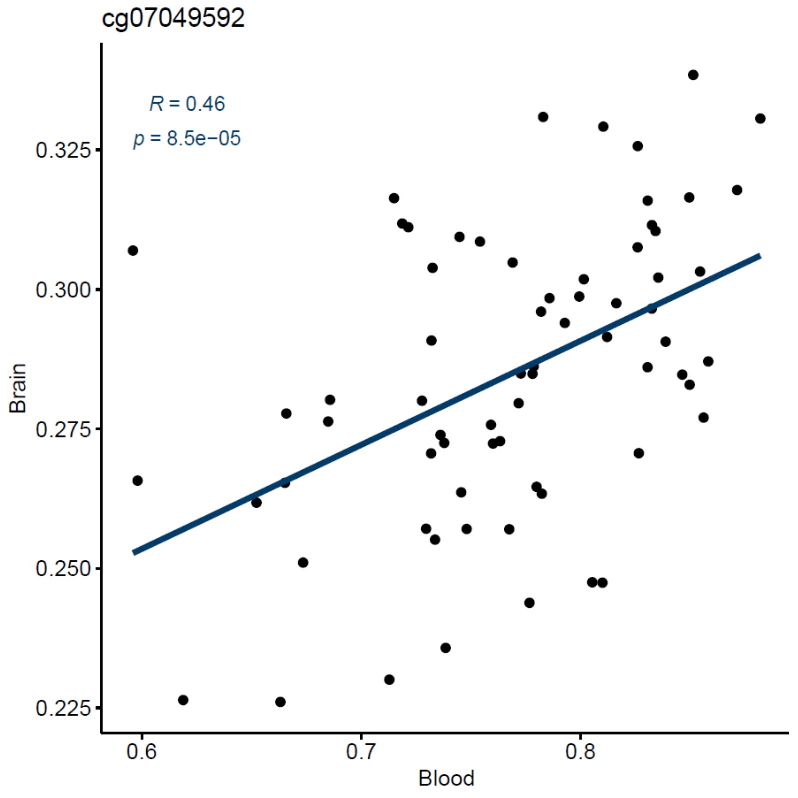
cg07049592
R = 0.46
p = 8.5e−05
0.325
0.300
0.275
0.250
0.225
Brain
0.6
0.7
0.8
Blood

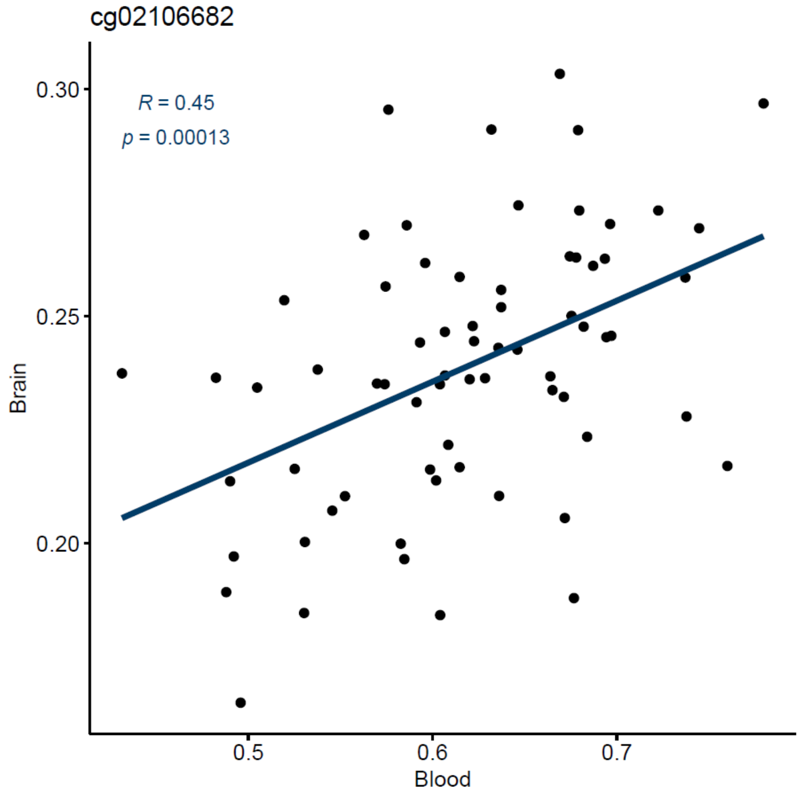
cg02106682
R = 0.45
p = 0.00013
0.30
0.25
0.20
Brain
0.5
0.6
0.7
Blood

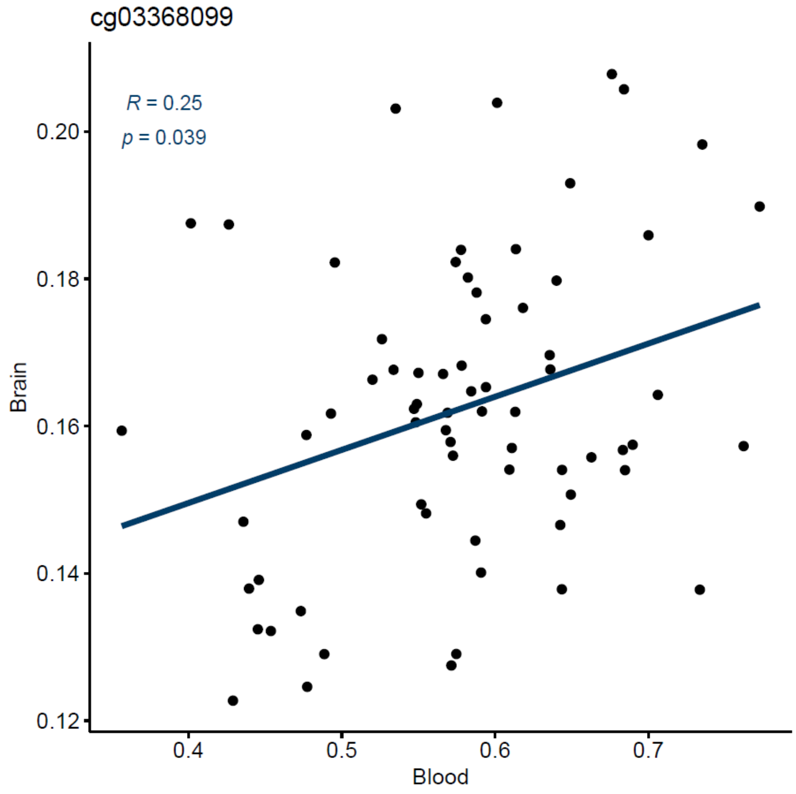
cg03368099
R = 0.25
p = 0.039
0.20
0.18
0.16
0.14
0.12
Brain
0.4
0.5
0.6
0.7
Blood

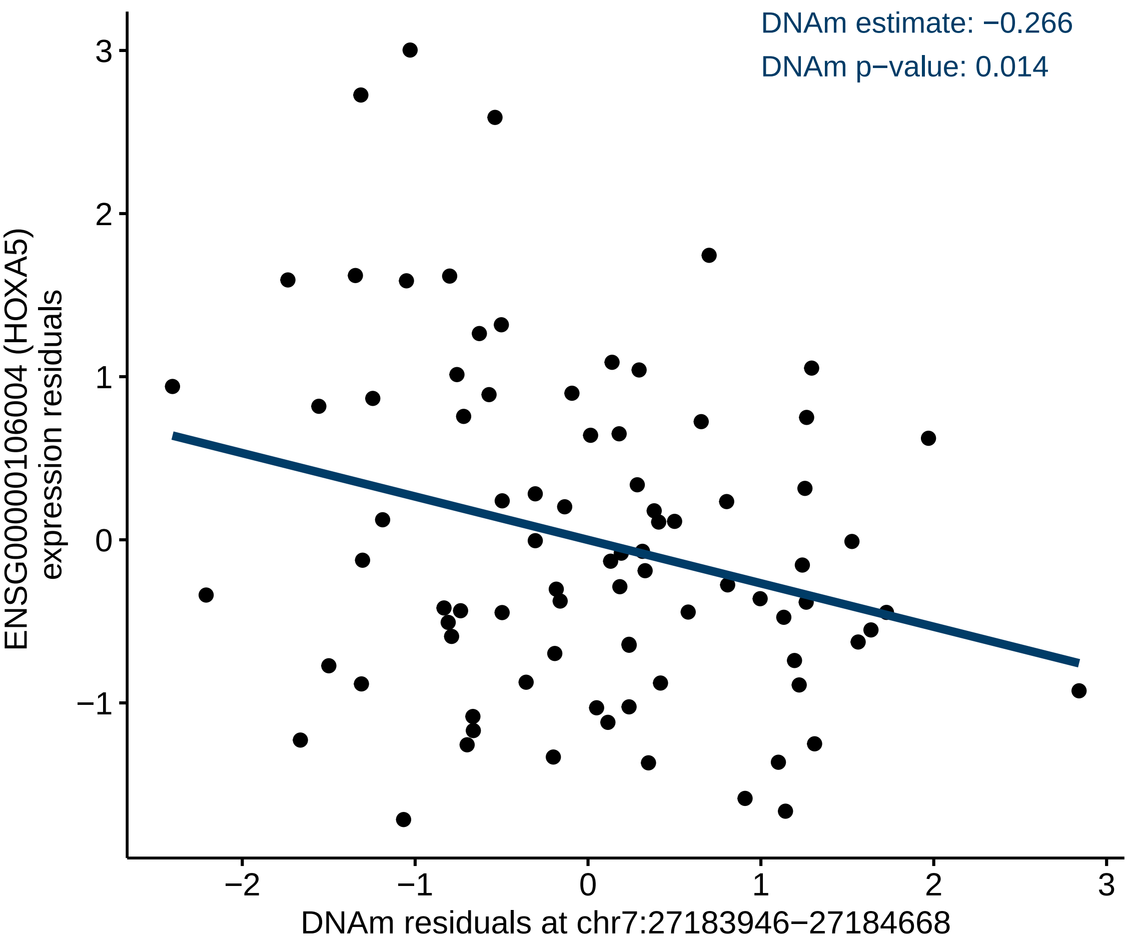

Supplementary Figure 8 DNA methylation (DNAm) at the DMR chr7:27183946 - 27184668 is significantly correlated with *HOXA5* gene expression. The ADNI dataset with matched gene expression and DNAm measured on 263 subjects was used for this analysis. Residuals were obtained by adjusting DNAm and gene expression separately by age, sex, immune cell-type proportions, batch effect, number of APOE4 alleles, smoking and years of education. Methylation level for the DMR was estimated by median methylation level over all CpGs mapped within the DMR.

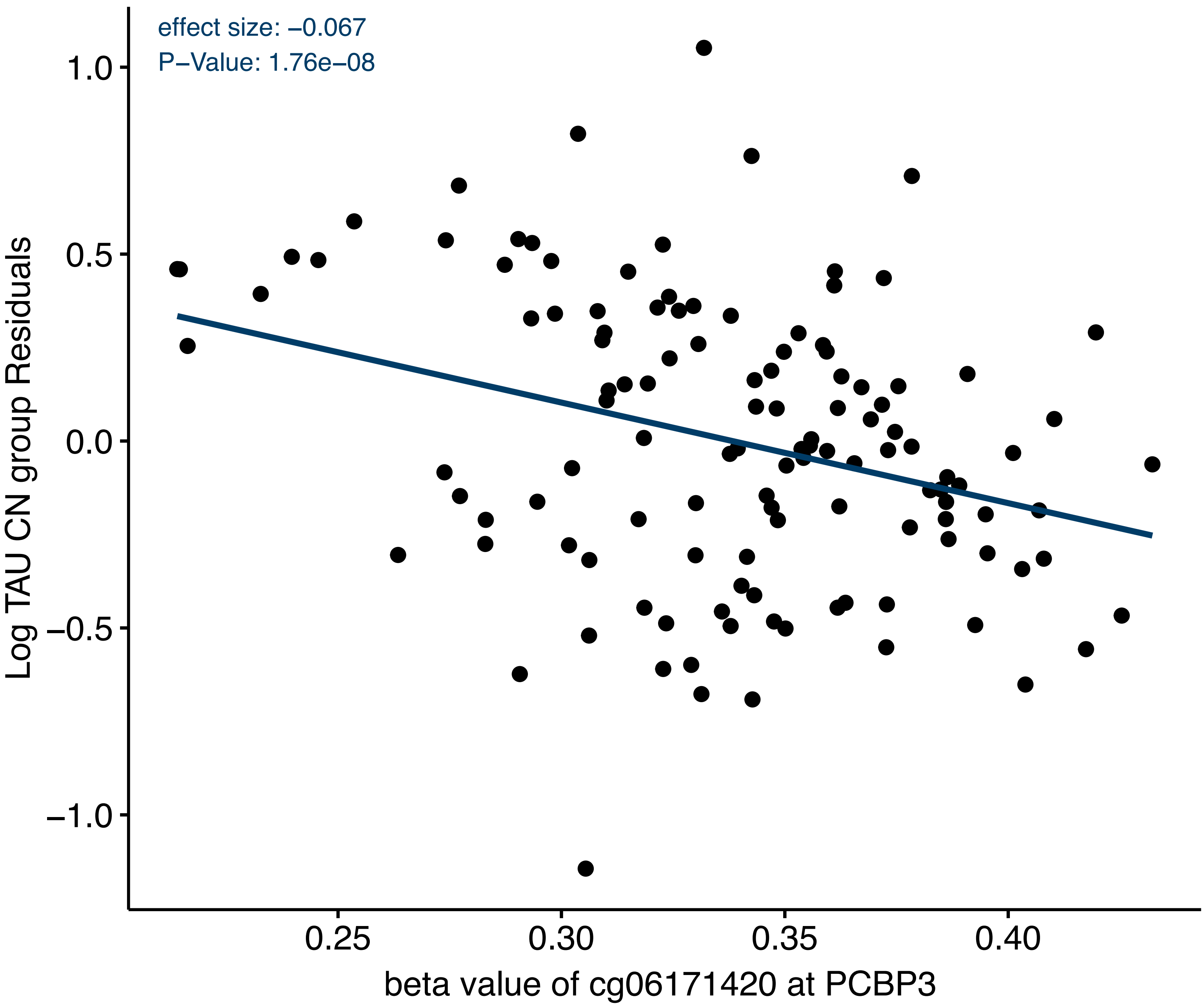

Supplementary Figure 9 DNA methylation at the CpG cg06171420 is significantly associated with CSF total tau (TAU) in cognitively normal samples in the ADNI dataset. To remove confounding effects from covariate variables, residuals were obtained by fitting model log CSF total tau ~ age + methylation plate + sex + APOE4 + years of education + smoking history + immune cell-type proportions.

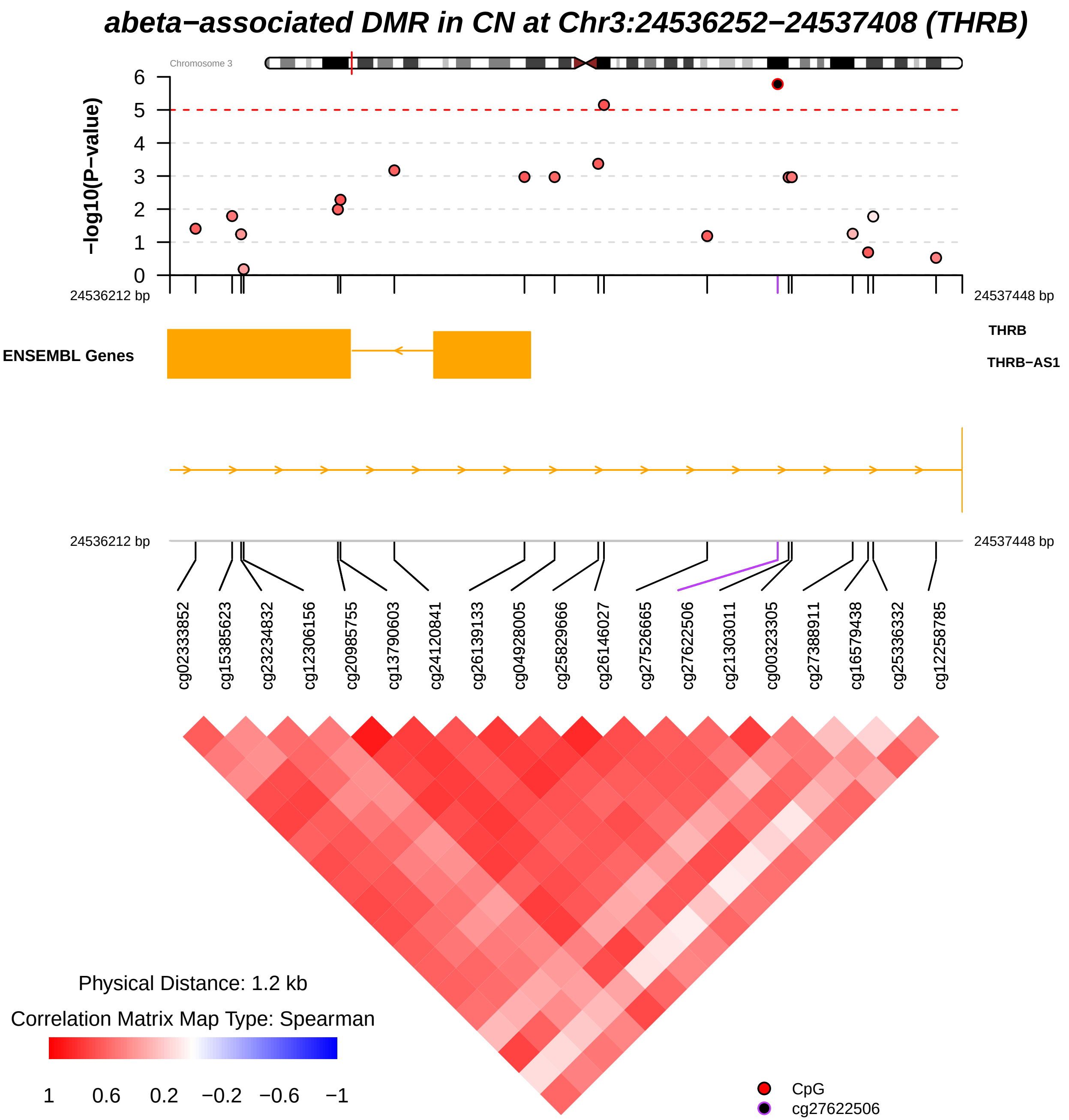

Supplementary Figure 10 Aβ_{42}-associated DMR on chromosome 3. The highlighted CpG next to purple line / purple dot is the most significant CpG in the DMR.

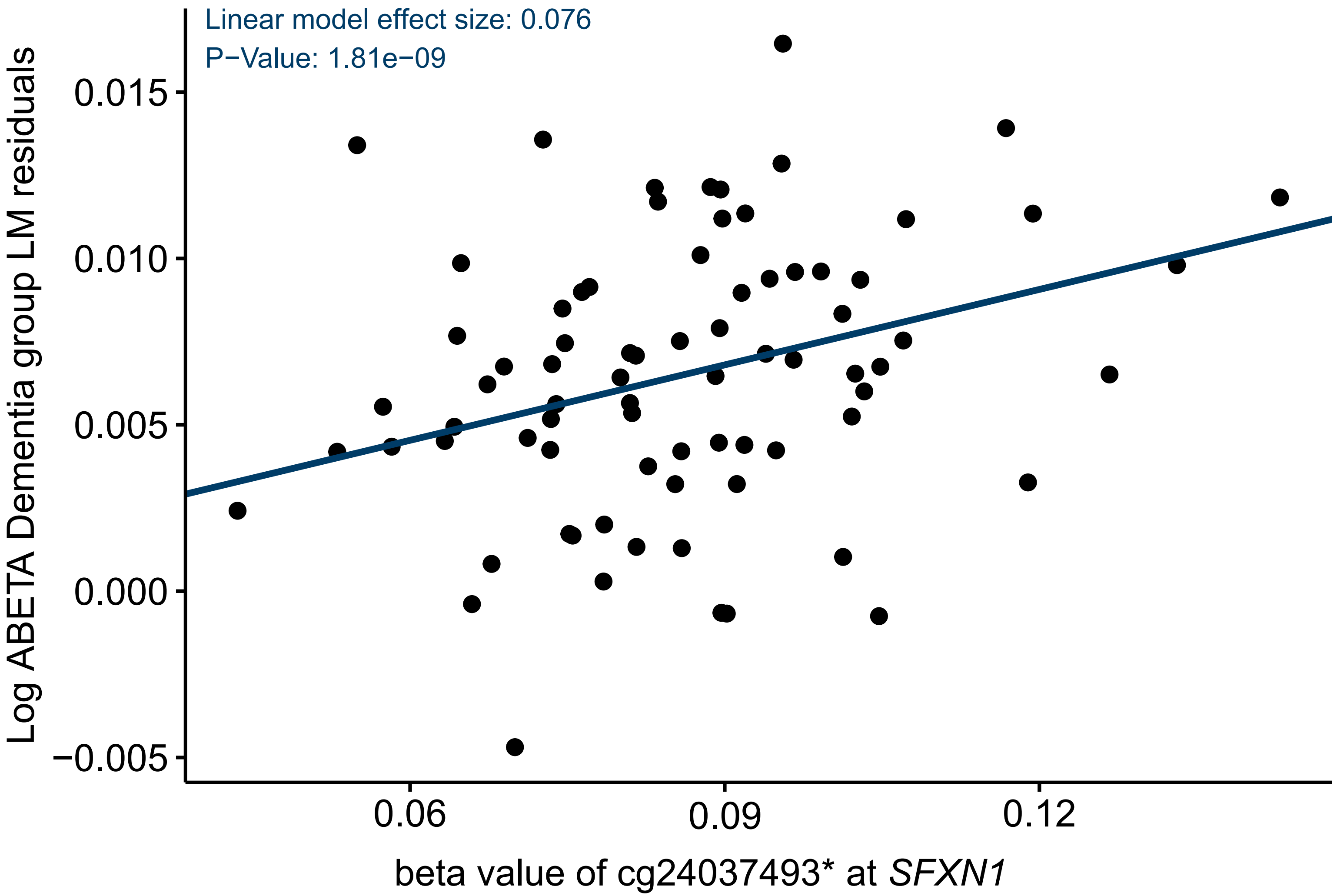

Supplementary Figure 11 DNA methylation at the CpG cg24037493 is significantly associated with CSF $A\beta_{42}$ in Alzheimer's disease subjects in the ADNI dataset. To remove confounding effects from covariate variables, residuals were obtained by fitting model log CSF $A\beta_{42}$ ~ age + methylation plate + sex + APOE4 + years of education + smoking history + immune cell-type proportions. *An outlier sample with beta value of 0.5, log abeta residual of 0.04 was omitted to improve resolution of the figure for all other data points. The fitted line represents estimated linear model applied to all data points, including the omitted outlier. Robust linear model, which is often used to model data with outlier, showed similar *P*-value for this CpG cg24037493 (*P*-value = 8.79×10^{-7}) as the *P*-value in linear regression (*P*-value = 1.81×10^{-9}).

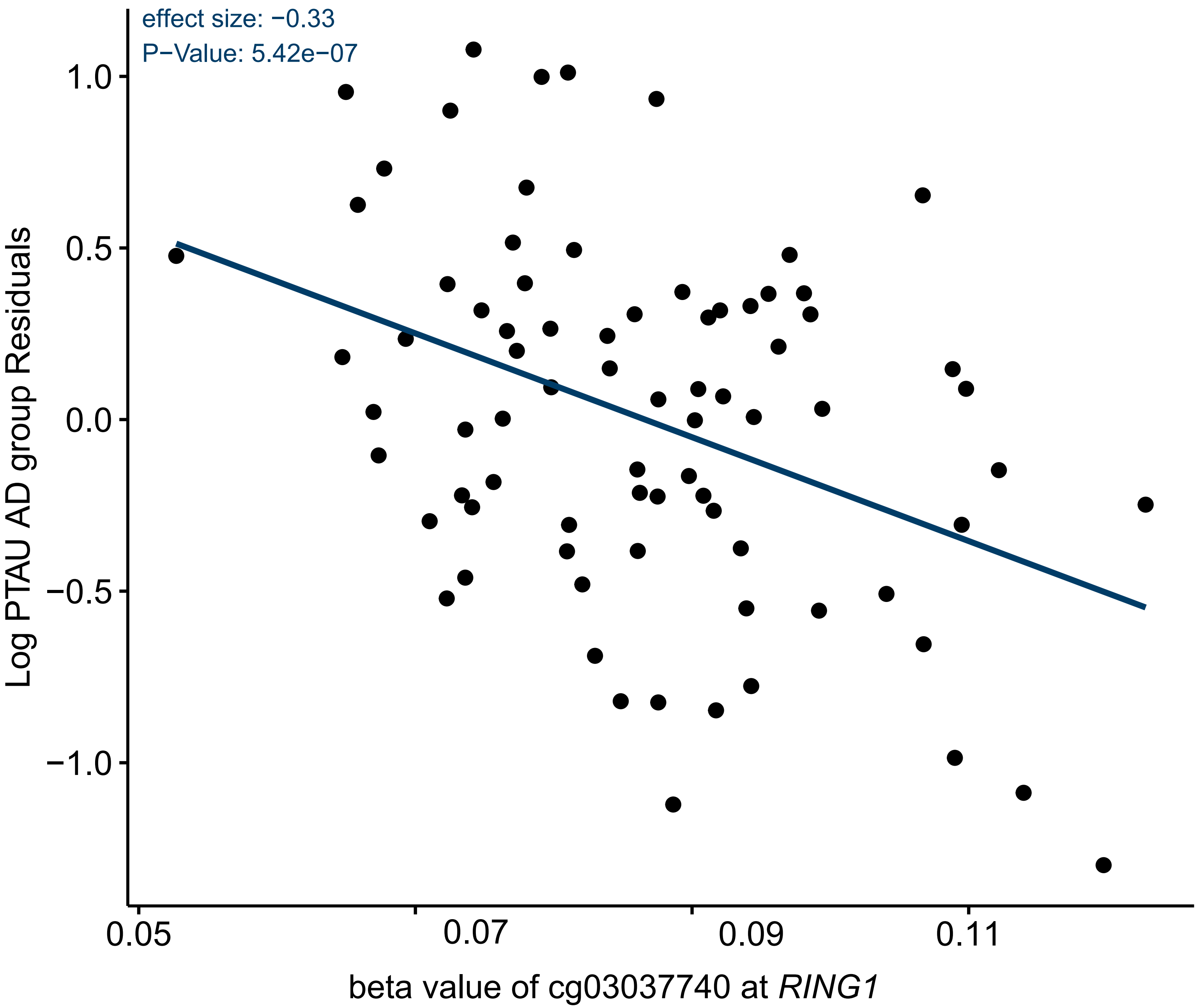

Supplementary Figure 12 DNA methylation at the CpG cg03037740 is significantly associated with CSF $pTau_{181}$ in Alzheimer's disease subjects in the ADNI dataset. To remove confounding effects from covariate variables, residuals were obtained by fitting model log CSF $pTau_{181}$ ~ age + methylation plate + sex + APOE4 + years of education + smoking history + immune cell-type proportions.

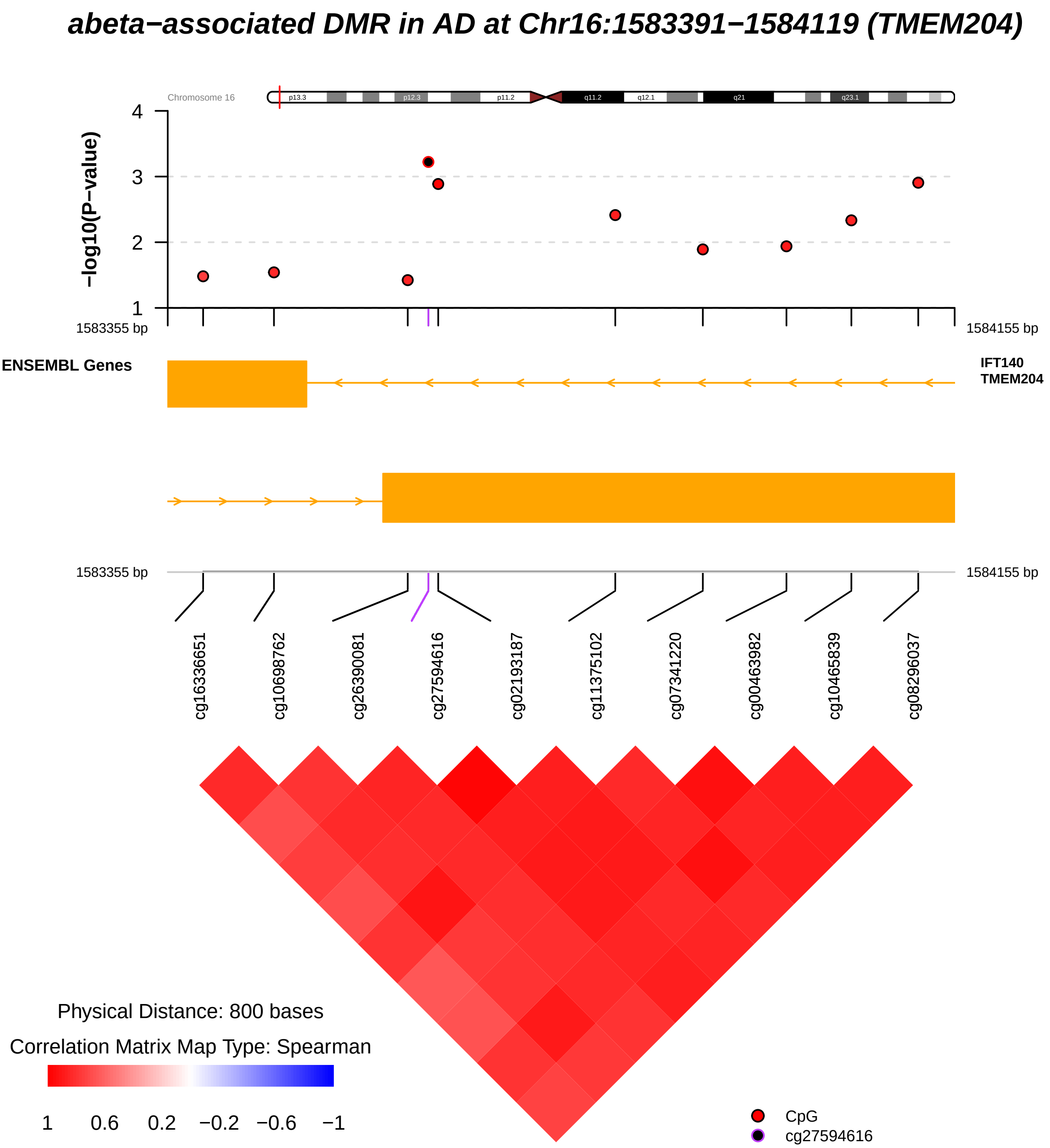

Supplementary Figure 13 The Aβ_{42}-associated DMR on chromosome 16. The highlighted CpG next to purple line / purple dot is the most significant CpG in the DMR.

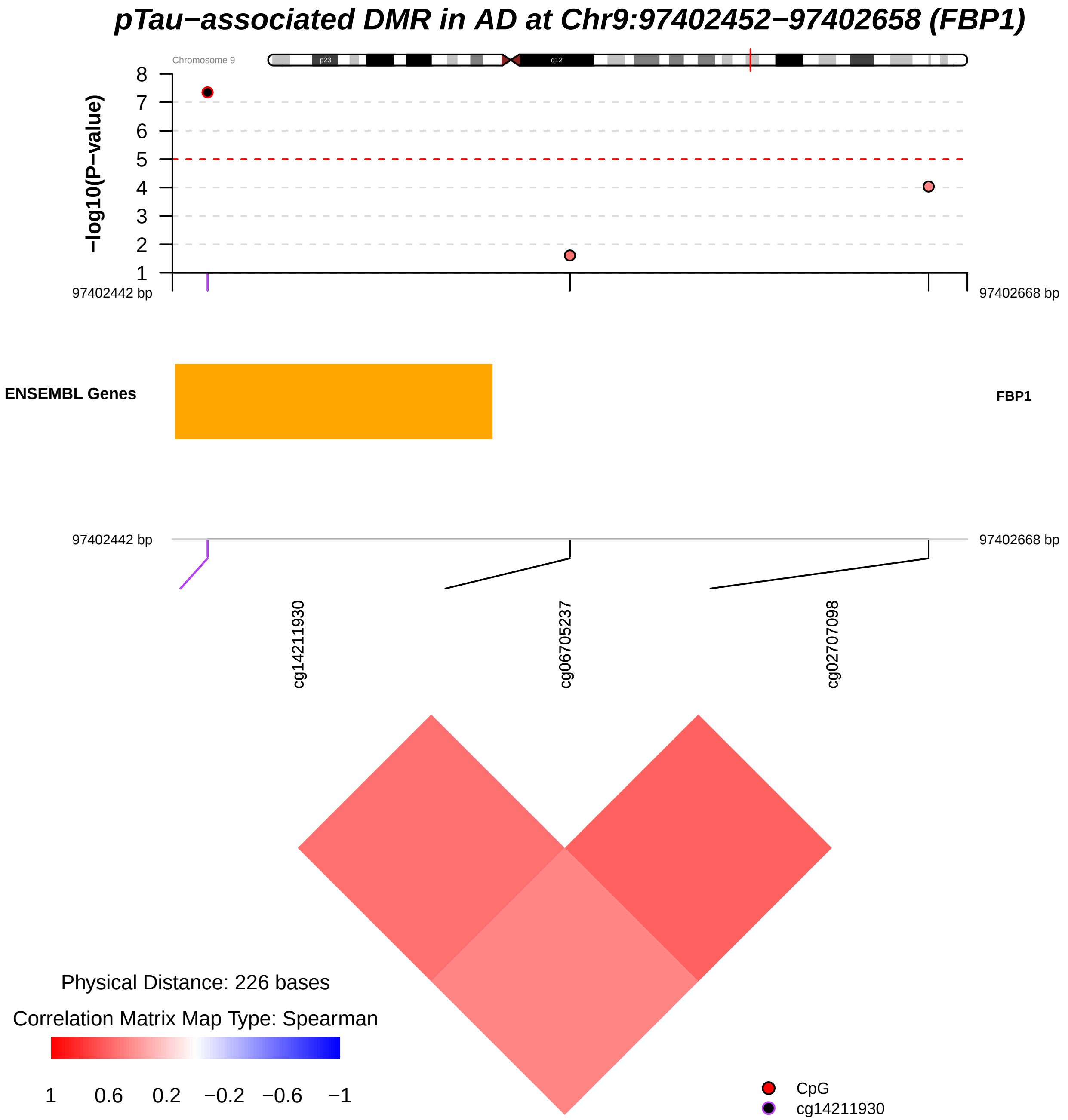

Supplementary Figure 14 The $pTau_{181}$-associated DMR on chromosome 9. The highlighted CpG next to purple line / purple dot is the most significant CpG in the DMR.

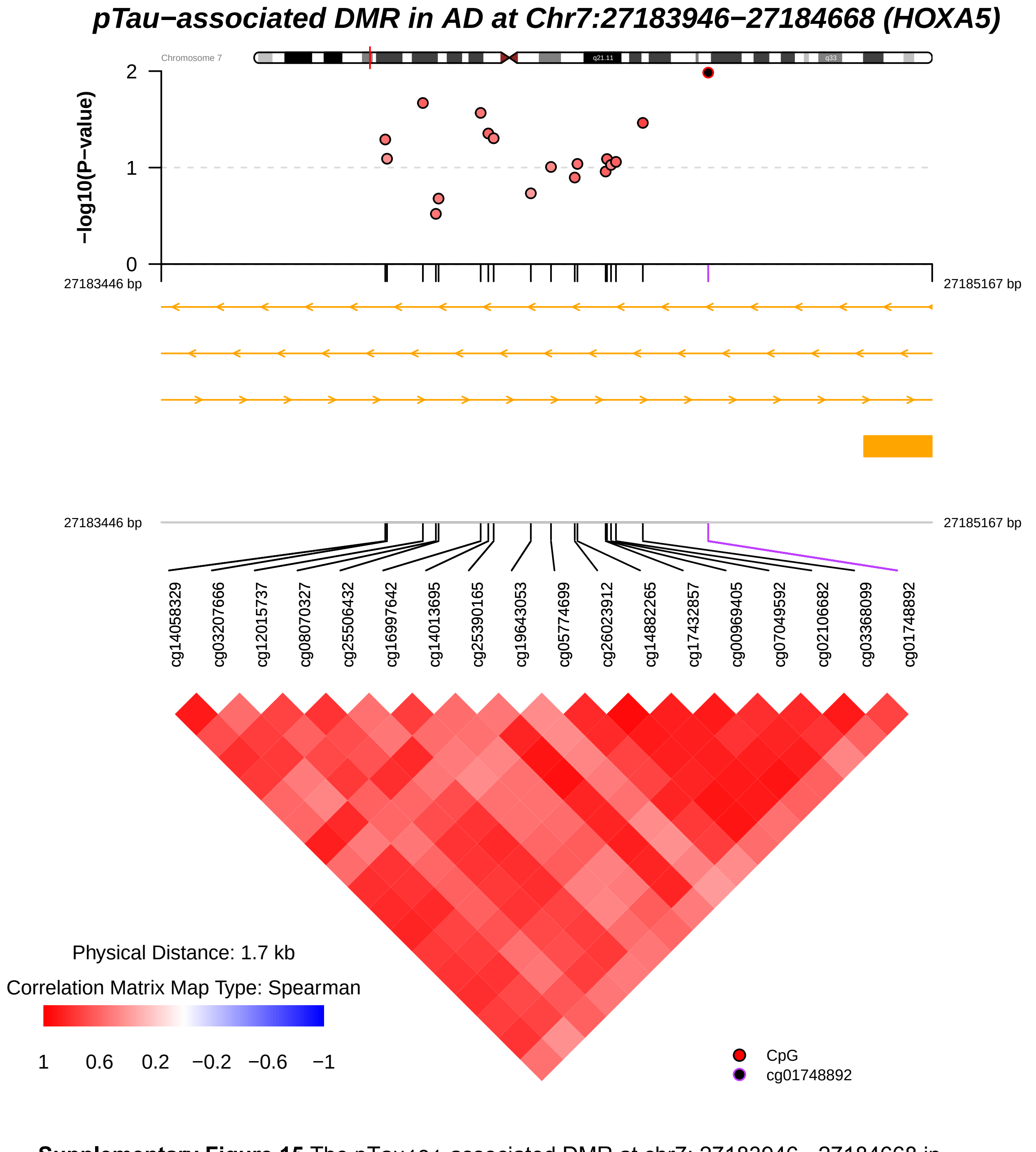

Supplementary Figure 15 The $pTau_{181}$-associated DMR at chr7: 27183946 - 27184668 in the ADNI dataset. The highlighted CpG next to purple line / purple dot is the most significant CpG in the DMR.
